# Factors affecting executive functions in obstructive sleep apnea syndrome and volumetric changes in the prefrontal cortex

**DOI:** 10.1186/s40064-016-3609-z

**Published:** 2016-11-08

**Authors:** Zahide Yılmaz, Nuray Voyvoda, Eda İnan, Pınar Bekdik Şirinocak, Rabia Terzi

**Affiliations:** 1 Clinic of Neurology, Kocaeli Derince Education and Research Hospital, Kocaeli, Turkey; 2 Clinic of Radiology, Kocaeli Derince Education and Research Hospital, Kocaeli, Turkey; 3 Clinic of Psychiatry, Kocaeli Derince Education and Research Hospital, Kocaeli, Turkey; 4 Clinical of Physical Medicine and Rehabilitation, Kocaeli Derince Education and Research Hospital, Kocaeli, Turkey

**Keywords:** Executive function, Prefrontal cortex, Obstructive sleep apnea, Excessive daytime sleepiness, Hypoxemia

## Abstract

**Purpose:**

Obstructive sleep apnea syndrome (OSAS) is associated with cognitive changes and executive functions are among the cognitive domains most affected. However, it is not completely understood which of the factor(s) among hypoxemia, repeated arousal, and sleepiness affect the executive functions. This study aims to evaluate the possible relationship between the executive functions and nocturnal parameters, Epworth Sleepiness Scale (ESS) scores, and prefrontal cortex (PFC) volumes.

**Patients and methods:**

A total of 28 patients aged between 18 and 60 years who were newly diagnosed with OSAS were included in this study. The Wisconsin Card Sorting Test (WCST) and Stroop test which were used in the evaluation of executive functions were applied to all patients. Cranial magnetic resonance imaging (MRI) and volumetric measurements of the PFC were performed. Polysomnography (PSG), WCST, Stroop test, and cranial MRI were also applied to the control group which consisted of age- and education status-matched 15 healthy subjects. The correlation of WCST and Stroop tests and PFC volume, PSG parameters, and ESS scale was examined.

**Results:**

The WCST-6 test scores were statistically significantly higher in the patient group (p = 0.022; p < 0.05). Additionally, the Stroop test 5 (p = 0.043) and Stroop test-5 correction (p = 0.005) measurements were statistically significantly higher in the patient group (p < 0.05). A negative and statistically significant correlation was found between the WCST-4 and WCST-10 and ESS measurements in the patient group (r −0.452; p 0.016; p < 0.05; r −0.437; p 0.020; p < 0.05). However, there was no correlation between the PSG parameters and WCST and Stroop test scores. No statistically significant differences in the MRI volumetric measurements of the PFC were found between the patient and control groups.

**Conclusions:**

Impairment in the attentive and executive functions in OSAS is evident. The most influential factor is excessive daytime sleepiness, rather than hypoxemia and severity of the disease.

**Electronic supplementary material:**

The online version of this article (doi:10.1186/s40064-016-3609-z) contains supplementary material, which is available to authorized users.

## Background

Obstructive sleep apnea syndrome (OSAS) is a common disorder of breathing during sleep characterized by repeated obstruction of the upper airway. The resolution of airway obstruction is accompanied by arousal from sleep (Borak et al. 1996).

Obstructive sleep apnea syndrome is accompanied by cognitive disorders. Among the cognitive domains most affected is the executive function (Saunamaki and Jehkonen 2007). Currently, the most widely accepted view is that neurocognitive impairment in OSAS is due to adverse effects of sleep fragmentation or intermittent hypoxia (Bucks et al. 2013). However, it is still unclear whether neurocognitive impairment is associated with excessive daytime sleepiness caused by chronic sleep fragmentation or with repeated apnea episodes. Sleep fragmentation may also mediate the cognitive deficits in OSAS via dysfunction in neural networks, particularly in the frontal lobes. The basis of the hypothesis is that sleep disruption reduces the efficacy of restorative processes in the prefrontal cortex (Horne 1998; Maquet 1995).

Beebe and Gozal defined the executive functions in six different subgroups as follows: behavioral inhibition, set-shifting, self-regulation of affection and arousal, working memory, analysis/synthesis, and contextual memory (Beebe and Gozal 2002). The Wisconsin Card Sorting Test (WCST) is the main tool to measure the executive functions (Baddeley et al. 1986; Lezak 1995; Pennington and Ozonoff 1996). Another widely used test to measure the executive functions is the Stroop test (Spreen and Strauss 2007). The WCST and Stroop tasks have been shown by functional magnetic resonance imaging (MRI) in neuroradiology to be associated with different regions of the prefrontal area; findings obtained under the Stroop test have been localized, particularly in the left frontal lobe, while the WCST performance has been localized mainly in the right frontal lobe (Karakaş and Karakaş 2000; Yaouhı et al. 2009).

In this study, we aimed to evaluate the executive functions in mild to severe OSAS and control groups using the WCST and Stroop test. Volumetric measurements of the prefrontal cortex (PFC) were performed by cranial MRI in the same patient group and we also investigated possible correlations of the findings with polysomnography (PSG) parameters and the Epworth Sleepiness Scale (ESS).

## Patients and methods

A written informed consent was obtained from each subject. The study protocol was approved by the local Ethics Committee. The study was conducted in accordance with the principles of the Declaration of Helsinki.

Patients who were newly diagnosed with OSAS at the Sleep and Sleep Disorders Laboratory of the Neurology Clinic at Kocaeli Derince Education and Research Hospital between 01.2012 and 12.2014 were included in the study. Patients aged between 18 and 60 years were evaluated. Patients with neurological diseases, mental disorders, severe psychiatric diseases, previous cancer, and other sleep disorders including central sleep apnea syndrome, periodic limb movement disorder, narcolepsy, and restless legs syndrome were excluded from the study. Patients were questioned for hypertension (HT), diabetes mellitus (DM), heart disease, and chronic obstructive pulmonary disease (COPD). The WCST and Stroop tests were performed in all patients by an experienced psychologist and cranial MRIs were obtained. A control group consisted of 15 age- and education status-matched healthy individuals.

The level of education of the patients were divided in three levels, as education duration of five years, between 8 and 12 years, and ≥13 years, defined as low, intermediate, and high education status, respectively.

The control group also received PSG, WCST, and Stroop tests, and cranial MRI were performed. Body mass index (BMI) was also calculated in all patients and controls. A full night PSG was performed in all patients and individuals of the control group at the sleep laboratory. The PSG included electroencephalography, electrooculography, chin and leg electromyography, electrocardiography, snoring, thermistor, nasal pressure transducer, finger pulse oximeter, thoracic and abdominal respiratory movements, and body position. Scoring was performed according to the 2010 American Academy of Sleep Medicine criteria. Patients with an Apnea/Hypopnea Index (AHI) of equal to or more than 5/h were accepted to have OSAS. An AHI value equal to or greater than 5/h and <15/h was defined as mild; an AHI value equal to or greater than 15/h and less than 30/h was defined as moderate, and an AHI value more than 30/h was defined as severe OSAS.

### Magnetic resonance imaging

Magnetic resonance imaging examinations of the patient and control groups were performed using the same MRI equipment (1,5 Tesla, Intera Master, Philips Medical Systems, USA) and a standard head spiral. In addition to the conventional MRI examination, A 2-mm section was obtained with inversion recovery (IR) placed at the coronal plane with the TSE method (TR/TE/TI: 2250/10/400 msn, NSA: 2, TSE factor 15). An IRTSE examination was used for volumetric measurements. All images were transferred to a CD and the measurements were performed on another computer by the same radiologist who was blind to the both patient and control groups.

**Fig. 1:**
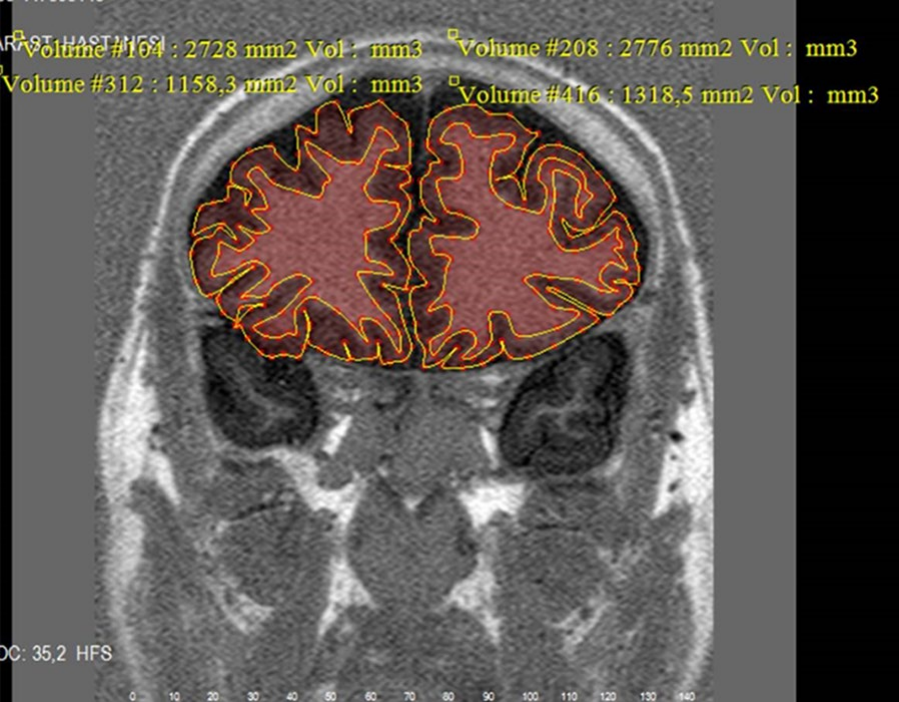
Areas of total and white matter of right and left prefrontal cortex were calculated

The right and left sides were evaluated separately in the prefrontal cortex. Sections from the anterior frontal lobe to the anterior genu of the corpus callosum were evaluated. The gray matter area was calculated using mathematical subtraction following the measurement of the area of the total PFC (white matter and gray matter) and the area of white matter (Fig. 1). These areas were multiplied by the thickness of the cross section, and volumetric data (raw data) was, then, obtained (Rademacher et al. 1992).

Since the magnitude of the head is variable in each individual, corrected volumes of those structures were calculated according to the formula used in the study by Watson et al. (1997) and Insausti et al. (1998).

$$ {\text{Corrected}}\;{\text{volume}} = \left[ {\left( {{\text{Mean}}\;{\text{intracranial}}\;{\text{area}}\;{\text{of}}\;{\text{all}}\;{\text{cases/the}}\;{\text{intracranial}}\;{\text{area}}\;{\text{of}}\;{\text{the}}\;{\text{case}}} \right) \times {\text{raw}}\;{\text{data}}\;{\text{volume}}\;{\text{of}}\;{\text{the}}\;{\text{case}}} \right] $$

For normalization, the section in which anterior commissure was present at the coronal IR sequence in the MRI examination of the patient and control groups was defined and the intracranial area of this level was measured. Intracranial area of all cases was calculated and divided into the intracranial area of the case. The calculation of this value with the raw data volume which was measured manually yielded the calculation of separate corrected volume values in each patient (Fig. 1).

### Neuropsychological examination

One of the main tests used in measuring the executive functions is the WCST (Karakaş and Karakaş 2000). Primarily, the perseveration and abstract scrutinizing; concept formation; defining property; attention; working memory; mental flexibility; problem solving; category creation; and changing categories are among the specifications which WCST is able to measure. The WCST, which is used as a frontal lobe test, is lateralized to the right hemisphere (Karakaş and Karakaş 2000) and has a distribution in the right frontal lobe including the dorsolateral PFC (Karakaş 2006). Normative data of the WCST were collected in the context of the Neuropsychological Test for Cognitive Potentials Battery. Two decks consisting of four stimulating cards and 64 test cards are included in the WCST test. The patient chooses a card from above the first deck and he/she should find the stimulating that card matches (matching is expected to be made according to the color, shape, or amount for two cycles) with the card he/she picks. While the instructions are given, no hints should be given to the patient about how and according to what the cards should be matched. Feedback should be given to the patient after each match on whether the matching is correct or not, and the patient should be expected to find the matching rule. The test is complete, when the patient completes six categories or when he/she uses all 128 cards. Scoring is performed according to a total of 13 categories, which are total number of answers, total number of erroneous answers, total number of correct answers, number of completed categories, perseverative responses, perseverative errors, non-perseverative errors, percentage of perseverative errors, number of trials used to find the first category, percentage of cognitive level response, points for failure to continue setup, point for learning how to learn, and points for failure to learn to learn (Karakaş 2006). In this study, the WCST-2 (total number of errors), WCST-4 (number of completed categories), WCST-6 (total number of perseverative errors), and WCST-10 (number of cognitive level responses) were included. When the patient insists on using previous principles and does not change the principle (perseveration), although the behavioral principle has been changed and he/she has received warning directions on this subject by the tester, it implies impairment in performance (Karakaş and Karakaş 2000).

In addition, we used the Stroop test, which is utilized to measure attention, in addition to executive functions. The Stroop test used in Turkey is prepared using the combination of original Stroop test and Victoria form of the Stroop test in the context of Neuropsychological Test for Cognitive Potentials Battery. Its reliability and validity tests were performed and normative data were collected. The form created as the Stroop test was called Basic Sciences Research Group (BSRG) Form (Karakaş 2006). The Stroop test measures the speed of data processing and attentive skills, mainly cognitive setup and responsive skills under destructive effects (Karakaş 2006).

There are four cards used in the Stroop test. In the first card, there are words of color names printed with black ink. The second card involves the same words of color names printed in different colors (e.g., the word ‘red’ printed with green ink), in the third card, there are colored circles and in the fourth card, there are neutral words (Turkish words; kadar, zayıf, ise, orta) printed in different colors.

During the test, the patient is asked to read the words of the color names printed in black in the first section (first card), to read the words of color names printed in different color, but not to tell the color of ink in the second section (second card), tell the names of the colors of colored circles in the third section (third card), tell the printed color of the word, but not to read the word (neutral Turkish words; kadar, zayıf, ise, orta) in the fourth section (fourth card). Finally, in the fifth section, the patient is asked to tell the printed the colors of the word, but not to read the word and tell red, but not green for the word ‘green’ printed in red-(second card again).

Impairment in performance is defined as lengthening of the duration of telling the colors with inability to resist the accustomed response (reading) or as telling the wrong color. Patients with a low resistance to interference and who are unable to cope with distractors read the article instead of telling the color. Spontaneous corrections and errors and time are recorded during scoring. Duration of reading the words printed in colored letters was subtracted from the duration of the section in which the patient told the colors, instead of reading the words. If this difference of duration is high and a high number of errors and spontaneous corrections is present, it indicates that the attention of the individual can be easily distracted and that the individual has difficulty in suppressing an inappropriate response tendency (Oktem 2006). The Stroop test performance is particularly associated with the left frontal lobe (Karakaş 2006). It is also quite sensitive to the frontal function disorders (Oktem 2006).

Furthermore, the patients were also evaluated using the ESS. It was used in the measurement of daily sleepiness condition of the patients (Karakoç et al. 2007; Izci et al. 2008). Patients with an ESS score >10 were considered to have excessive daytime sleepiness.

### Statistical analysis

The NCSS (Number Cruncher Statistical System) 2007 (Kaysville, Utah, USA) software was used for statistical analysis. Descriptive data were expressed in mean, standard deviation, median, frequency, percentage, and minimum and maximum values. The Mann–Whitney U test was used to compare two groups without normal distribution, while the Kruskal–Wallis test was used to compare the quantitative variables of three groups. Qualitative variables were also compared using the Pearson’s Chi square and Fisher’s exact tests. The Spearman’s correlation analysis was used to analyze correlations between the variables. *p* values of <0.01 and <0.05 were considered statistically significant.

## Results

Of a total of 43 subjects, 26 (60.5%) were males and 17 (39.5%) were females with a mean age of 44.53 ± 8.08 (range 28–60) years (Table 1). There was no statistically significant difference in the mean age, sex distribution, and education status between the patient and control groups (p > 0.05). The BMIs of the cases in the patient group were statistically significantly higher, compared to the controls (p = 0.007; p < 0.01). In addition, ESS scores were statistically significantly higher in the patient group, compared to the control group (p < 0.05). The incidence of HT, cardiac disease, COPD, and DM was similar between the patient and control groups (p > 0.05). The AHI and Time SO_2_ < 90% measurements were also statistically significantly higher in the patient group, compared to the control group (p = 0.001; p < 0.01). However, min SO_2_% measurements were statistically significantly lower in the patient group, compared to the control group (p = 0.001; p < 0.01). There was no statistically significant difference in the non-rapid eye movement (NREM)1%, NREM2%, NREM3%, and rapid eye movement (REM) % measurements between the groups (p > 0.05) (Table 2). No statistically significant differences were found in the PFC right total, white matter, gray matter (subtraction) and PFC left total, and white matter and gray matter (subtraction) MRI volumetric measurements (p > 0.05) (Table 3).

**Table 1:** Demographic characteristics of patient and control groups

	Total (n = 43)	Patient (n = 28)	Control (n = 15)	p
Age (years)				
Min–max (median)	28–60 (45)	32–60 (46)	28–57 (40)	0.054^a^
BMI (kg/m^2^)				
Min–max (median)	21.50–44.30 (31.3)	22.80–44.30 (32.5)	21.50–41.40 (26)	0.007^a,^ **
Sex				
Male	26 (60.5)	19 (67.9)	7 (46.7)	0.176^b^
Female	17 (39.5)	9 (32.1)	8 (53.3)	
Duration of education (years)				
Min–max (median)	1–15 (7)	1–12 (6)	5–15 (8)	0.178
Education status				
Low education	22 (51.2)	16 (57.1)	6 (40.0)	
Intermediate education	17 (39.5)	12 (42.9)	5 (33.3)	
High education	4 (9.3)	0	4 (26.7)	
ESS				
Min–max (median)	0–19 (10)	1–19 (11.5)	0–14 (6)	0.036*

*BMI* body mass index

** p < 0.01; * p < 0.05

^a^Mann–Whitney U-test

^b^Pearson’s Chi square test

^c^Fisher’s exact test

**Table 2:** Polysomnographic findings of patient and control groups

	Total (n = 43)	Patient (n = 28)	Control (n = 15)	p^a^
AHI (/h)				
Min–max (median)	0.50–93.10 (16.5)	6.50–93.10 (30.8)	0.50–4.30 (2.3)	0.001**
NREM1 (%)				
Min–max (median)	1.80–25.10 (5.6)	1.80–13.70 (5.05)	2.30–25.10 (6.3)	0.198
NREM2 (%)				
Min–max (median)	2.70–70 (22)	4.20–70 (20.05)	2.70–52.70 (35.4)	0.386
NREM3 (%)				
Min–max (median)	13.70–81.20 (49.1)	15.10–74.70 (49.7)	13.70–81.20 (37)	0.241
REM (%)				
Min–max (median)	0.90–24.30 (13.4)	4.30–24.30 (12.8)	0.90–23.80 (15.7)	0.516
Time SO_2_ < 90% (%)				
Min–max (median)	0–29.60 (0.2)	0–29.60 (0.9)	0–0.40 (0)	0.001**
Min SO_2_ (%)				
Min–max (median)	61–95 (85)	61–89 (83)	85–95 (93)	0.001**

*AHI* Apnea/Hypopnea Index, *REM* rapid eye movement, *NREM* non-rapid eye movement

** p < 0.01

^a^Mann–Whitney U-test

**Table 3:** Magnetic resonance imaging volumetric measurements of patient and control groups^3^

	Group			P
	Total (n = 43)	Patient (n = 28)	Control (n = 15)	
PFC RT (mm^3^)				
Min–max (median)	39,693–74,861 (58,662)	39,693–70,979 (58,321)	45,404–74,861 (59,453.28)	0.300
PFC RWM (mm^3^)				
Min–max (median)	12,317–28,286 (19,073)	12,317–28,286 (19,036)	13,258–25,432 (19,073)	0.585
PFC RGM (subtraction) (mm^3^)				
Min–max (median)	27,376–49,429 (39,450)	27,376–46,554 (38,927)	32,146–49,429 (39,987)	0.152
PFC LT (mm^3^)				
Min–max (median)	36,005–77,029 (61,300)	36,005–77,029 (58,552)	42,237–74,080 (61,300)	0.365
PFC LWM (mm^3^)				
Min–max (median)	11,746–31,214 (20,281)	11,746–31,214 (19,999)	13,868–25,385 (21,171)	0.553
PFC LGM (subtraction) (mm^3^)				
Min–max (median)	24,023–48,694 (40,357)	24,023–48,209 (40,289)	28,368–48,694 (40,921)	0.348

Student’s *t* test

*PFC* prefrontal cortex, *RT* right total, *RWM* right white matter, *RGM* right gray matter, *LT* left total, *LWM* left white matter, *LGM* left gray matter

The WCST-2 and WCST-10 test measurements were higher in the patient group; WCST-4 was lower in the patient group and *p* values were close to statistical significance. However, no statistically significant difference was found between the two groups (p > 0.05). The WCST-6 test was statistically significantly higher in the patient group (p = 0.022; p < 0.05). In the patient group, the Stroop test-5 (p = 0.043) and Stroop test-5 correction (p = 0.005) measurements were also statistically significantly higher, compared to the control group (p < 0.05). No statistically significant differences were found in the Stroop test-5 error measurements (p > 0.05) (Table 4).

**Table 4:** Wisconsin Card Sorting Test and Stroop Test scores of patient and control groups

	Group			P
	Total (n = 43)	Patient (n = 28)	Control (n = 15)	
WCST-2				
Mean ± SD	52.72 ± 24.15	57.68 ± 24.25	43.47 ± 21.79	0.061
Min–max (median)	7–92 (55)	7–92 (61)	11–78 (54)	
WCST-4				
Mean ± SD	3.72 ± 1.77	3.36 ± 1.78	4.40 ± 1.59	0.052
Min–max (median)	0–6 (3)	0–6 (3)	1–6 (4)	
WCST-6				
Mean ± SD	22.86 ± 17.99	27.18 ± 19.02	14.80 ± 12.89	0.022*
Min–max (median)	0–82 (20)	0–82 (22)	0–45 (11)	
WCST-10				
Mean ± SD	47.28 ± 19.79	43.36 ± 18.44	54.60 ± 20.77	0.061
Min–max (median)	6–87 (50)	13–74 (42.5)	6–87 (58)	
Stroop test difference between 1–5				
Mean ± SD	19.47 ± 8.78	21.18 ± 8.76	16.27 ± 8.17	0.061
Min–max (median)	4–40 (18)	4–39 (20)	7–40 (16)	
Stroop test-5				
Mean ± SD	30.07 ± 9.80	32.25 ± 10.10	26.00 ± 7.99	0.043*
Min–max (median)	15–55 (28)	15–55 (30)	16–49 (24)	
Stroop test-5 error				
Mean ± SD	0.77 ± 1.32	0.82 ± 1.19	0.67 ± 1.59	0.174
Min–max (median)	0–5 (0)	0–4 (0)	0–5 (0)	
Stroop test-5 correction				
Mean ± SD	1.51 ± 1.53	1.86 ± 1.46	0.87 ± 1.51	0.005**
Min–max (median)	0–6 (1)	0–5 (1.5)	0–6 (1)	

Mann–Whitney U-test

*WCST* Wisconsin Card Sorting Test

* p < 0.05; ** p < 0.01

Furthermore, no statistically significant difference was found in the MRI volumetric measurements of the PFC right total, white matter, gray matter (subtraction), PFC left total, and white matter and gray matter (subtraction) according to the disease severity (p > 0.05) (Additional file 1: Table S1). No statistically significant differences in the WCST-2, 4, 6, and 10 measurements and Stroop test difference between-1–5, Stroop test-5, Stroop test error, and Stroop test-5 correction measurements of the cases according to the severity of the disease and MRI volumetric measurements were observed (p > 0.05) (Table 5; Additional file 2: Table S2).

**Table 5:** Wisconsin Card Sorting Test and Stroop Test scores based on disease severity

	Group			p
	Mild (n = 6)	Moderate (n = 8)	Severe (n = 14)	
WCST-2				
Mean ± SD	51.0 ± 25.91	58.38–31.04	60.14 ± 20.42	0.690
Min–max (median)	18–79 (54.5)	7–88 (67.5)	19–92 (61)	
WCST-4				
Mean ± SD	3.83 ± 1.83	3.25 ± 2.25	3.21 ± 1.57	0.773
Min–max (median)	2–6 (3.5)	0–6 (3)	1–6 (3)	
WCST-6				
Mean ± SD	28.17 ± 20.58	26.25 ± 18.15	27.29 ± 20.26	0.945
Min–max (median)	5–60 (27.5)	0–62 (22)	4–82 (21.5)	
WCST-10				
Mean ± SD	51.0 ± 18.69	33.88 ± 17.99	45.5 ± 17.61	0.215
Min–max (median)	26–73 (58.5)	15–60 (28)	13–74 (47)	
Stroop test difference between 1–5				
Mean ± SD	19.67 ± 9.79	23.25 ± 9.99	20.64 ± 8.05	0.716
Min–max (median)	4–31 (21.5)	8–39 (22.5)	13–35 (17)	
Stroop test-5				
Mean ± SD	29.33 ± 10.67	36.63 ± 8.26	31.00 ± 10.68	0.272
Min–max (median)	15–42 (30.5)	25–48 (36.5)	20–55 (27.5)	
Stroop test-5 error				
Mean ± SD	0.50 ± 0.55	0.38 ± 0.74	1.21 ± 1.48	0.492
Min–max (median)	0–1 (0.5)	0–2 (0)	0–4 (1)	
Stroop test-5 correction				
Mean ± SD	1.33 ± 1.03	2.13 ± 1.46	1.93 ± 1.64	0.298
Min–max (median)	0–3 (1)	1–5 (1.5)	0–5 (2)	

Kruskal–Wallis Test

*WCST* Wisconsin Card Sorting Test

In addition, there was no statistically significant difference in the ESS results according to the disease severity (p > 0.05) (Additional file 3: Table S3). No statistically significant correlations were found between the WCST-2, 4, 6, and 10 and Time SO_2_ < 90% and min SO_2_ measurements in the patient group (p > 0.05) (Table 6).

**Table 6:** Epworth Sleepiness Scale scores, Min SO_2_ and time SO_2_ < 90% values by Wisconsin Card Sorting Test and Stroop Test in the patient group

Patient group (n = 28)	ESS		Min SO_2_ (%)		Time SO_2_ < 90 (%)	
	r	p	R	p	r	p
WCST-2	0.370	0.053	0.232	0.234	0.072	0.715
WCST-4	0.452	0.016*	0.213	0.277	0.070	0.722
WCST-6	0.126	0.522	0.044	0.823	0.024	0.904
WCST-10	0.437	0.020*	0.102	0.606	0.077	0.697
Stroop test 1–5 difference	0.087	0.660	0.052	0.793	−0.026	0.895
Stroop test-5	0.014	0.944	−0.034	0.862	0.005	0.978
Stroop test-5 error	0.223	0.254	0.002	0.991	0.137	0.488
Stroop test-5 correction	−0.193	0.325	−0.165	0.402	0.079	0.691

*r* Spearman’s Correlation coefficient, *WCST* Wisconsin Card Sorting Test

* p < 0.05

On the other hand, we found a negative and statistically significant correlation between the WCST-4 and WCST-10 and ESS measurements (r −0,452; p 0.016; p < 0.05; r −0.437; p 0.020; p < 0.05) (Table 6). However, no statistically significant correlation was found between the Stroop test-1–5, Stroop test-5, Stroop test% error, Stroop test-5 correction measurements, and ESS, Min SO_2_ and Time SO_2_ < 90% measurements (p > 0.05) (Table 6).

## Discussion

Obstructive sleep apnea syndrome has been associated with a broad range of psychological problems and neurocognitive difficulties, particularly in memory and new learning, attention, and executive function, which are the most widely reported. Among the cognitive domains, executive function is the most affected domain (Saunamaki and Jehkonen 2007). However, whether neurocognitive impairment is associated with excessive daytime sleepiness caused by chronic sleep fragmentation or recurrent apnea episodes still remains to be elucidated. The aim of the current study is to contribute the understanding of the main causes of impairment of executive functions in OSAS. Impaired performance in OSAS has been reported in several tests of various cognitive functions, such as attention, vigilance, memory, psychomotor performance, and executive functioning (Aloia et al. 2004). Among those functions, recently, the most riveting is the executive function. Neuropsychologists define the executive function as a flexible approach under problematic conditions and maintenance and development of an organized condition (Dencla 1996; Eslinger 1996). Executive functions allow individuals to use their basic skills in a complex and variable environment in a coherent way (Eslinger 1996; Goldberg 2001). In the present study, we used the Stroop test to evaluate the focused attention, reaction inhibition, and speed of data processing and the WCST test to evaluate working memory, abstract thinking, perseveration, conceptualization, and executive functions. The WCST-6 measurements were significantly higher in the patient group. It demonstrated that identity mapping skills in the patient group were particularly lower and that they had difficulty in actualization of changing response behavior which was expected, despite the instructions which were given.

Additionally, the Stroop test-5 and Stroop test-5 correction measurements were higher in the patient group, compared to the control group. This finding indicated that patients had more difficulty in the skills of changing the perceptional setup under distracting effect, inhibiting accustomed behavior patterns, and focused attention, compared to the control group. As a result, we observed problems in complicated attention and executive functions in the patient group. In addition, OSAS is accompanied by impairment in several cognitive domains, including attention and vigilance decrements, memory gaps, and abnormalities in executive functions (Saunamaki and Jehkonen 2007; Kim et al. 1997; Ferini-Strambi et al. 2003). The findings of the present study are consistent with the previous findings.

Furthermore, factors contributing to cognitive dysfunctions are not completely understood, yet (Beebe and Gozal 2002). The present study found a negative correlation between the WCST-4 and ESS measurements. The patients with higher ESS scores were observed to define a less number of categories, compared to patients with lower ESS scores (p = 0.016). This finding likely indicated that these patients had more difficulty in identity mapping and conceptual reasoning. In addition, it demonstrated that these patients had more difficulty in the expected changing response behavior, despite the instructions which were given, compared to the patients with lower ESS scores. Additionally, a negative correlation was observed between the WCST-10 and ESS measurements. The number of patients with higher ESS scores with the number of conceptual responses were statistically significantly lower than the patients with lower ESS scores (p = 0.020). This likely indicated that these patients had more difficulty in learning rules, maintaining matching, and conceptual reasoning, compared to the patients with lower ESS scores and also demonstrated that the working memory of these patients was weaker.

However, we found no correlation between the WCST and Stroop subgroups and Time SO_2_ < 90% and Min SO_2_ measurements. In a study by Shpirer et al. (2012), the authors found no correlation between the executive functions and sleep parameters. However, attention, AHI, and hypoxemia parameters (mean spO2 and percent time spent with a SpO2 < 90%) were correlated, although no association of these with the severity of sleepiness was detected. Furthermore, in another study by Bucks et al. (2013), sleep fragmentation was found to have a more strong effect on attention and vigilance, compared to hypoxemia (Bucks et al. 2013). Consistent with our study findings, Torelli et al. (2011) also found no correlation between neuropsychological functions and respiratory data. In another study, both excessive daytime sleepiness and nocturnal hypoxemia were found to equally contribute to cognitive deficits (Engleman and Joe 1999; Engleman et al. 2000).

In contrast to our findings, Bedard et al. (1991) and Cheshire et al. (1992) reported that dysfunction in executive functions was correlated with blood gas abnormalities and sleep interruption, rather than excessive daytime sleepiness. We also found that excessive daytime sleepiness more affected the impairment of attention and executive function, rather that hypoxemia and AHI.

Furthermore, patients need excess sleep the day after due to sleep interruptions secondary to repeated apnea, hypopnea, and arousals during sleep (Schlosshan and Elliott 2004; Douglas and Polo 1994). Daytime excessive sleepiness is the most commonly seen symptom among patients with OSAS (Karakoç et al. 2007; Mediano et al. 2007; Banno and Kryger 2007). However, not all patients with OSAS complain of daytime sleepiness. Pathophysiological causes of excessive daytime sleepiness are not completely understood to date. The level of the complaints of daytime sleepiness may individually vary in patients who share the same demographic characteristics and the same AHI values. The current study did not find any relationship between the disease severity of OSAS and ESS. The mechanisms of this condition are still unclear (Roure et al. 2008). On the other hand, the severity of sleepiness may not be associated with the severity of the disease (Banno and Kryger 2007). Mediano et al. (2007) reported that patients with daytime sleepiness had shorter sleep latencies, increased sleep effectivity, and poor nocturnal oxygenation, compared to individuals who did not have daytime sleepiness. Although the association of OSAS and daytime excessive sleepiness has been known for a long time, daytime cognitive and behavioral dysfunction, which seems to be beyond accompanying simple sleepiness, has been documented with the behavioral research conducted for the past two decades (Arens 2000; Marrone 1998).

In the patient group of this study, no correlation was detected between the disease severity of OSAS and attentive and executive functions. In a review by Bucks et al. (2013), five published articles were analyzed and disease severity was not associated with cognitive functions in three of them. Also, in another study by Borges et al. (2013), no significant correlation was found between AHI and executive performance. These findings are also consistent with our study findings.

The PFC serves for special executive functions (Bradley and Floras 2000). Disorders associated with OSAS affect PFC-related cognitive functions during the restorative phase of sleep (Beebe and Gozal 2002). In a study by Beebe and Gozal (2002), dysfunction in the PFC of the brain was demonstrated in patients with OSAS. In addition, PFC dysfunction was demonstrated to be associated with sleep disorders in several studies (Harrison and Horne 1997, 1998, 1999, 2000). These functional differences are related to the structural tissue damage and metabolic stress in various brain tissue compartments. On the other hand, previous neuroimaging studies performed in patients with OSAS demonstrated controversial results. In the present study, PFC volume measurements were similar in the patient and control groups, and had no correlation with the disease severity. However, MRI was inadequate to demonstrate the overt cerebral damage in a study conducted by Davies et al. (2001). Functional MRI studies may show occult neuronal dysfunction better than morphological MRI. Positron emission tomography (PET) study performed by Yaouhi et al. (2009) showed reduced PFC metabolism. Also, reduced activity in the prefrontal gyri was detected by functional MRI in a study by Zhang et al. (2011). In a review published in 2006, Zimmermen and Aloia (2006) reported either the absence of activation in the dorsolateral PFC or increased neuronal response in the frontal lobe, when a cognitive task was applied in functional neuroimaging cognitive studies. Hence, more sensitive and quantitative MRIs seem to be more successful in demonstrating the specific regions of the brain in cognitive functions in patients with OSAS, rather than volumetric MRI studies.

Functional magnetic resonance imaging (MRI) studies in neuroradiology demonstrated that the WCST and Stroop tasks are associated with different regions of the prefrontal region. Findings obtained under the Stroop task can be localized mainly in the left frontal lobe, and WCST success mainly in the right frontal lobe (Karakaş and Karakaş 2000; Yaouhı et al. 2009). In this present study, although no difference was found in the PFC volumes between the patient and control groups, a negative correlation close to statistical significance was achieved between the PFC left total volumetric measurement and Stroop test-5 correction test results in the patient group. A correlation close to statistical significance was also observed between the PFC left white matter volumetric measurement and Stroop test-5 correction test results. Of note, we performed the measurements with structural volumetric MRI, while previous studies were carried out using the functional MRI. However, we believe that this finding is invaluable to demonstrate the correlation between the Stroop test and left frontal lobe and also is consistent with the previous findings in terms of localization.

## Conclusion

In conclusion, impairment in executive functions is evident in OSAS. The most influential factor affecting this impairment is excessive daytime sleepiness, rather than hypoxemia and severity of the disease. Functional MRI which is more sensitive and more quantitative seems to be more successful in the visualization of specific regions of the brain, rather than volumetric MRI in cognitive functions in patients with OSAS.

## Additional files

**Additional file 1: Table S1.** Evaluation of MRI volumetric measurements by disease severity.

**Additional file 2: Table S2.** Comparison of MRI volumetric measurements and neuropsychological tests in the patient group.

**Additional file 3: Table S3.** Evaluation of ESS by disease severity.
