# Supplementary material for: Factors affecting executive functions in obstructive sleep apnea syndrome and volumetric changes in the prefrontal cortex
Source: Springerplus. 2016 Nov 8;5(1):1934. doi: 10.1186/s40064-016-3609-z (PMC5101245; doi:10.1186/s40064-016-3609-z)
Supplement: Supplementary file 1 — Additional file 1: Table S1. Evaluation of MRI volumetric measurements by disease severity. [file 40064_2016_3609_MOESM1_ESM.docx]

**Additional file 1: Table S1. Magnetic resonance imaging volumetric measurements based on disease severity**

|  | **Disease Severity**  Mild (n=6)   Moderate (n=8)  Severe (n=14)  p | | | | |
| --- | --- | --- | --- | --- | --- |
| **PFC RT (mm^3^)** | *Mean±SD* | 57184±7411 | 55997±4264 | 59899±8909 | *0.341* |
|  | *Min-Max(Median)* | 47958-69510 (56755) | 47721-60444 (57367) | 39693-70979 (63336) |  |
| **PFC RWM (mm^3^)** | *Mean±SD* | 18496±3891 | 18012±2664 | 19510±4610 | *0.627* |
|  | *Min-Max(Median)* | 13259-23311 (18995) | 13811-21805 (18203) | 12317-28286 (20153) |  |
| **PFC RGM (subtraction) (mm^3^)** | *Mean±SD* | 38687±3945 | 36768±4365 | 40389±4973 | *0.113* |
|  | *Min-Max(Median)* | 34699-46199 (37853) | 28250-41548 (38378) | 27376-46554 (41793) |  |
| **PFC LT (mm^3^)** | *Mean±SD* | 56615±7102 | 58558±10436 | 59455±12289 | *0.683* |
|  | *Min-Max(Median)* | 51512-67233 (52570) | 46869-77029 (58552) | 36005-76826 (63858) |  |
| **PFC LWM (mm^3^)** | *Mean±SD* | 18221±3976 | 19707±5321 | 19862±5806 | *0.817* |
|  | *Min-Max(Median)* | 12550-22970 (17741) | 13639-29662 (19379) | 11746-31214 (21696) |  |
| **PFC LGM (Subtraction) (mm^3^)** | *Mean±SD* | 38394±4458 | 38851±5329 | 39593±7174 | *0.767* |
|  | *Min-Max(Median)* | 32254-45113 (37843) | 33120-47366 (38203) | 24023-48209 (40939) |  |

Kruskal-Wallis test. PFC, prefrontal cortex; RT, right total; RWM, right white matter; RGM, right gray matter; LT, left total; LWM, left white matter; LGM, left gray matter.
