# Supplementary material for: Factors affecting executive functions in obstructive sleep apnea syndrome and volumetric changes in the prefrontal cortex
Source: Springerplus. 2016 Nov 8;5(1):1934. doi: 10.1186/s40064-016-3609-z (PMC5101245; doi:10.1186/s40064-016-3609-z)
Supplement: Supplementary file 2 — Additional file 2: Table S2. Comparison of MRI volumetric measurements and neuropsychological tests in the patient group. [file 40064_2016_3609_MOESM2_ESM.docx]

Additional file2: Table S2. Magnetic resonance imaging volumetric measurements and neuropsychological tests in the patient group

|  | | **WCST-2** | | **WCST-4** | | **WCST-6** | | **WCST-10** | | **STROOP TEST DIFFERENCE BETWEEN 1-5** | | **STROOP TEST-5** | | **STROOP TEST-5 Error** | | **STROOP TEST-5 Correction** | |
| --- | --- | --- | --- | --- | --- | --- | --- | --- | --- | --- | --- | --- | --- | --- | --- | --- | --- |
|  |  | **r** | ***p*** | **r** | ***p*** | **r** | ***p*** | **r** | ***p*** | **r** | ***p*** | **r** | ***p*** | **r** | ***p*** | **r** | ***p*** |
| **Patient group (n=28)** | **PFC RT (mm^3^)** | 0.141 | ***0.474*** | 0.217 | ***0.268*** | 0.088 | ***0.657*** | 0.166 | ***0.398*** | 0.024 | ***0.904*** | 0.024 | ***0.903*** | 0.288 | ***0.137*** | 0.126 | ***0.521*** |
|  | **PFC RWM (mm^3^)** | 0.232 | ***0.236*** | 0.308 | ***0.111*** | 0.029 | ***0.882*** | 0.209 | ***0.285*** | 0.039 | ***0.844*** | 0.056 | ***0.775*** | 0.317 | ***0.100*** | 0.141 | ***0.475*** |
|  | **PFC RGM (Subtraction) (mm^3^)** | 0.097 | ***0.624*** | 0.174 | ***0.375*** | 0.164 | ***0.405*** | 0.208 | ***0.288*** | 0.019 | ***0.924*** | 0.045 | ***0.818*** | 0.220 | ***0.261*** | 0.134 | ***0.496*** |
|  | **PFC LT (mm^3^)** | 0.135 | ***0.493*** | 0.073 | ***0.711*** | 0.094 | ***0.633*** | 0.075 | ***0.705*** | 0.095 | ***0.630*** | 0.157 | ***0.425*** | 0.229 | ***0.241*** | 0.342 | ***0.075*** |
|  | **PFC LWM (mm^3^)** | 0.164 | ***0.404*** | 0.132 | ***0.503*** | 0.124 | ***0.528*** | 0.033 | ***0.867*** | 0.066 | ***0.737*** | 0.164 | ***0.406*** | 0.238 | ***0.223*** | 0.275 | ***0.156*** |
|  | **PFC LGM (Subtraction) (mm^3^)** | 0.019 | ***0.923*** | 0.041 | ***0.835*** | 0.045 | ***0.818*** | 0.103 | ***0.601*** | 0.110 | ***0.578*** | 0.187 | ***0.341*** | 0.135 | ***0.494*** | 0.370 | ***0.053*** |

^r:^ Spearman’s correlation coefficient, *p<0.01. PFC, prefrontal cortex; RT, right total; RWM, right white matter; RGM, right gray matter; LT, left total; LWM, left white matter; LGM, left gray matter.
