# Supplementary material for: Factors affecting executive functions in obstructive sleep apnea syndrome and volumetric changes in the prefrontal cortex
Source: Springerplus. 2016 Nov 8;5(1):1934. doi: 10.1186/s40064-016-3609-z (PMC5101245; doi:10.1186/s40064-016-3609-z)
Supplement: Supplementary file 3 — Additional file 3: Table S3. Evaluation of ESS by disease severity. [file 40064_2016_3609_MOESM3_ESM.docx]

Additional file 3: Table S3. Epworth Sleepiness Scale based on disease severity

|  | | **Disease Severity** | | | **p** |
| --- | --- | --- | --- | --- | --- |
|  |  | **Mild (n=6)** | **Moderate (n=8)** | **Severe (n=14)** |  |
| **ESS** | *Mean±SD* | 9.17±5.84 | 9.50±6.26 | 11.79±5.42 | ***0.521*** |
|  | *Min-Max (Median)* | 1-16 (8.5) | 2-18 (8.5) | 2-19 (12) |  |

Kruskal-Wallis Test. ESS, Epworth Sleepiness Scale.
